# Photon-Counting Detektoren – ein Quantensprung für die Computertomographie?

**DOI:** 10.1016/j.zemedi.2022.05.001

**Published:** 2022-06-08

**Authors:** Martin Pichotka

**Affiliations:** Freiburg, Deutschland

Die Computertomographie (CT) hat sich über Jahrzehnte hinweg als zuverlässiges Arbeitspferd der klinischen Bildgebung bewährt. Dank der großen Robustheit, gepaart mit hoher räumlicher und zeitlicher Auflösung, hat sich diese Bildgebungsmodalität in der klinischen Diagnostik des 20ten Jahrhunderts schnell etabliert und ist mittlerweile im klinischen Alltag unersetzlich geworden. Auch in der Materialforschung, der Qualitätskontrolle, der Archäologie und in vielen weiteren Feldern findet CT-Bildgebung breite Anwendung. Gegenüber anderen, nicht-destruktiven Analysemethoden zeichnet sich die CT insbesondere dadurch aus, dass sie sehr niedrige Ansprüche an die zu untersuchenden Proben stellt. Seit ihrer Entwicklung durch Hounsfield hat sich die klinische CT-Bildgebung erheblich weiterentwickelt; wesentliche Meilensteine dieser Evolution waren insbesondere das Spiral-CT [1] und das Dual-Energy CT [2], [3] – beide Entwicklungen liegen jedoch schon einige Jahrzehnte zurück.

In jüngerer Zeit zeichnet sich erneut ein starkes wissenschaftliches Interesse an der Entwicklung von CT-Methoden ab. Hierbei steht eine neue Detektortechnologie, der so genannte photonenzählende Detektor (*photon counting detector*, PCD), im Zentrum des Interesses. PCDs sind eine Klasse der direkt konvertierenden Halbleiterdetektoren, deren Technologie ihren Ursprung in der Hochenergiephysik hat, in der sie zur Verfolgung von Trajektorien ionisierender Teilchen verwendet werden. In den 1990er Jahren wurde am CERN die Medipix-Kollaboration gegründet mit dem Ziel, diese Detektortechnologie aus der Hochenergiephysik in die Medizin und andere Anwendungsfelder zu transferieren. Die PCD-Technologie stieß weltweit auf großes wissenschaftliches Interesse und wurde in einer Vielzahl von Anwendungsfeldern erprobt und fortlaufend optimiert. Mittlerweile gibt es jenseits der Medipix-Kollaboration eine Reihe kommerzieller Anbieter photonenzählender Detektoren. Erste klinische Systeme mit PCD-Technologie sind in jüngster Zeit ebenfalls verfügbar geworden.

Doch was macht die PCD-Technologie so revolutionär? Dies zu verstehen erfordert einen Einblick in die Funktionsweise der Detektoren.

Ein direkt konvertierender Halbleiterdetektor besteht aus einem Halbleitersensor mit einem hohen intrinsischen Widerstand, der mit einer anwendungsspezifischen integrierten Schaltung (*application-specific integrated circuit*, ASIC) verbunden ist. Die ionisierende Röntgenstrahlung erzeugt im Halbleitersensor freie Ladungsträger (Elektronen und „Löcher“), die mit Hilfe eines elektrischen Feldes separiert werden, wo sie abhängig vom Vorzeichen der Ladung entweder zum Rückseitenkontakt des Sensors oder zur pixelierten Oberfläche des ASIC transportiert werden. Hier werden die Ladungsträger mittels analoger und digitaler Elektronik prozessiert – jeder Detektorpixel des ASICs enthält zu diesem Zweck eine komplexe Elektronik, mit der eingehende Ladungspulse analysiert und gespeichert werden können. Eine schnelle Taktung dieser Elektronik (z.B. mit 640 MHz bei Medipix3) erlaubt die separate Analyse der Signale einzelner Teilchen. Hierin besteht ein wesentlicher Unterschied zu klassischen Röntgendetektoren, die die Signale vieler Photonen über einen definierten Zeitraum integrieren. Sowohl in Szintillatoren als auch in Halbleitersensoren ist die Anzahl generierter Sekundärteilchen (Sekundärphotonen bzw. Ladungsträger) näherungsweise proportional zu der Wechselwirkungsenergie. Klassische Röntgendetektoren summieren jedoch die Energie der eingetroffenen Photonen und werden deshalb, im Gegensatz zu photonenzählenden Detektoren, als Energie-integrierend bezeichnet.

Die Prozessierung eingehender Signale in Echtzeit durch einen ASIC erlaubt eine hohe Konfigurierbarkeit der Detektoren. So lässt sich zum Beispiel Detektorrauschen durch Dunkelströme oder elektronisches Rauschen unterdrücken, indem ein Schwellwert gesetzt wird, oberhalb dessen ein Signal überhaupt verwertet wird. Durch Verwendung mehrerer Schwellwerte kann zudem spektrale Information auch bei hohen Photonenflüssen gewonnen werden. Darüber hinaus kann auch eine komplexere Signalprozessierung in die Pixellogik implementiert werden einschließlich Methoden, die Information aus benachbarten Pixeln verwenden. So verfügt zum Beispiel der Medipix3-ASIC [4] über eine Ladung-addierende Schaltung (*charge summing circuit*), die eine Zuweisung der Energiebeiträge von Nachbarpixeln auf den Pixel mit dem höchsten Energiebeitrag erlaubt. Hierdurch wird die Verbreiterung der Ladungsträgerwolke während des Transports durch den Sensor, das so genannte *Charge-Sharing*, kompensiert, so dass sich gleichzeitig eine hohe räumliche und spektrale Auflösung realisieren lässt.

Auch die Signalpropagation im Halbeitersensor eines PCDs unterscheidet sich von der in einem konventionellen Szintillator, wie er in klassischen CT Systemen verwendet wird. Während in einem Szintillator sekundäre Photonen erzeugt werden, die sich isotrop ausbreiten, werden die Ladungsträger im PCD durch das angelegte elektrische Feld geleitet. Dies impliziert einen hohen intrinsischen Kontrast und erlaubt die Verwendung tiefer Sensoren mit hoher Quanteneffizienz. Die Notwendigkeit der Segmentierung des Sensors, die insbesondere bei geringen Pixelgrößen zu signifikantem Verlust an aktivem Volumen und damit an Detektionseffizienz führt, entfällt bei PCDs wegen des günstigen Transportverhaltens der Ladungsträger.

Die Fähigkeit der PCDs zur spektral aufgelösten Röntgenbildgebung bei hohen Photonenflüssen kann in der CT-Bildgebung eingesetzt werden, um Gewebe mit unterschiedlichen Absorptionseigenschaften zu diskriminieren. Dieses Konzept findet bereits in der Dual-Energy CT (DECT) Bildgebung Anwendung, bei der CT-Daten mit zwei unterschiedlichen Röntgenenergien aufgenommen werden. Die DECT ist jedoch auf die Diskriminierung zweier Gewebeklassen beschränkt. Auch führt die typischerweise starke Überlappung der Spektren bei DECT zu einer eingeschränkten Diskriminierungsfähigkeit von Geweben mit ähnlichen Absorptionseigenschaften. Dennoch bietet die DECT-Bildgebung deutliche Vorteile gegenüber konventioneller CT-Bildgebung und kann als Indikator für die potenziellen Vorteile spektraler CT-Bildgebung verstanden werden. So macht zum Beispiel die Fähigkeit der DECT-Bildgebung, Iod von Weichteilgewebe zu unterscheiden, eine native CT-Aufnahme ohne Kontrastmittel überflüssig und erlaubt dadurch eine signifikante Dosisreduktion [5]. In der spektralen PCD-CT-Bildgebung können darüber hinaus mehrere Materialien und Gewebearten simultan diskriminiert werden. So können insbesondere die Strahlaufhärtungseffekte, die zu einer Vielzahl von Bildartefakten führen, durch eine akkurate Materialdekomposition sehr gut unterdrückt werden. Zusätzlich werden neue CT-Kontrastmittel entwickelt, die auf schweren Elementen basieren und dadurch im spektralen CT gut detektierbare Absorptionskanten erzeugen, so dass die diagnostischen Fähigkeiten der PCD-CT-Bildgebung in den kommenden Jahren voraussichtlich stark erweitert werden.

Jenseits der technischen Vorteile der PCD-Technologie führt deren Etablierung in der klinischen Bildgebung auch zu merklichen Synergieeffekten. Insbesondere der direkte Kontakt zur physikalischen Grundlagenforschung ist ein starker Innovationstreiber für die CT-Bildgebung. So kann zum Beispiel die Optimierung von Tomographendesigns und die Entwicklung neuer bildgebender Methoden durch Verwendung von Simulationswerkzeugen aus der Grundlagenforschung stark beschleunigt und verbessert werden [GATE [6]) und Allpix2 [7]].

Gleichzeitig führen neue Entwicklungen in der Signalverarbeitung (*compressed sensing* [8], iterative Volumenrekonstruktion, Analysemethoden aus dem Bereich des Maschinellen Lernens) zusammen mit zunehmender Rechenleistung durch GPU-Computing zu vielen Verbesserungen der Bildqualität in der CT. Auch wenn diese Verbesserungen bereits dem konventionellen CT zugute kommen, so ermöglichen die von PCDs gelieferten, wesentlich differenzierteren Spektral-CT-Daten neue Analyseansätze. Hierbei entstehen allerdings auch neue Herausforderungen, da sich die multispektralen Daten nicht vollständig durch Grauwerte repräsentieren lassen – so müssen z.B. durch Materialsegmentierung und optimierte Darstellung Wege gefunden werden, diese Daten in einer für den Menschen verständlichen Weise aufzubereiten.

Während die letzten Jahrzehnte eher von graduellen Verbesserungen der CT-Technologie im Rahmen proprietärer Entwicklungen geprägt waren, ist jetzt zu erwarten, dass es wieder zu einer Zunahme universitärer Forschung im Rahmen des Generationenwechsels zur PCD-Technologie kommt, und dass neue Analysemethoden nicht nur die CT-Bildgebung befruchten werden.

Die Photon-Counting Technologie und die damit verbundenen Synergieeffekte stellen also durchaus einen Quantensprung in der CT-Bildgebung dar. Die vollständige Beherrschung der Technologie sowie die Entwicklung neuer Methoden für die spektrale CT-Bildgebung wird jedoch noch einige Zeit in Anspruch nehmen.
